# A prospective epidemiological study of new incident GISTs during two consecutive years in Rhône Alpes region: incidence and molecular distribution of GIST in a European region

**DOI:** 10.1038/sj.bjc.6605743

**Published:** 2010-06-29

**Authors:** P A Cassier, F Ducimetière, A Lurkin, D Ranchère-Vince, J-Y Scoazec, P-P Bringuier, A-V Decouvelaere, P Méeus, D Cellier, J-Y Blay, I Ray-Coquard

**Affiliations:** 1 Department of Medicine, Centre Léon Bérard, Lyon, France; 2 EA4129 ‘Santé, Individu, Société’ Centre Léon Bérard, Lyon, France; 3 Department of Pathology, Centre Léon Bérard, Lyon, France; 4 Department of Pathology, Hôpital Edouard Herriot, Hospices Civils de Lyon, Lyon, France; 5 Université de Lyon, Université Claude Bernard Lyon 1, Villeurbanne, France; 6 Department of Surgery, Centre Léon Bérard, Lyon, France; 7 Merck Serono, Lyon, France; 8 INSERM U590, Centre Léon Bérard, Lyon, France

**Keywords:** gastrointestinal stromal tumour (GIST), incidence, KIT, PDGFRA, kinase mutations

## Abstract

**Background:**

Preliminary data indicate that the molecular epidemiology of localised gastrointestinal stromal tumour (GIST) may be different from that of advanced GIST. We sought to investigate the molecular epidemiology of sarcomas, including GIST, in the Rhone-Alpes region in France.

**Patients and methods:**

A prospective and exhaustive study in the Rhone-Alpes Region in France to assess the precise incidence of primary sarcomas with systematic centralised pathological review and molecular analysis was conducted for 2 consecutive years.

**Results:**

Among 760 patients with a confirmed diagnosis of sarcoma, 131 (17%) had a GIST. The majority of patients had gastric primaries (61%). Mutational analysis could be performed in 106 tumour samples (74%), and 71 (67%) had exon 11 mutations. *PDGFRA* mutations were found in 16% of cases, which is twice as high as previously reported for advanced GIST.

**Conclusion:**

Data indicate that *PDGFRA* mutations in localised GIST may be twice as high as what was previously reported in patients with advanced disease. This finding may have important consequences for patients offered adjuvant imatinib, although most of these tumours are in the low-risk group.

Gastrointestinal stromal tumours (GISTs) are rare tumours of mesenchymal origin arising in the gastrointestinal tract. Incidence data obtained from registries indicate an incidence of approximately 12–15 new cases per million inhabitants per year in western countries (Goettsch *et al*, 2005; Nilsson *et al*, 2005); however, there remain uncertainties because of variations in diagnostic criteria before the NCI consensus reported in 2002 (Fletcher *et al*, 2002). Approximately 95% of GISTs stain positive for CD117 (KIT) and 85% of cases harbour activating mutations in the gene of one of two structurally related transmembrane tyrosine-kinase receptors: KIT and PDGFRA (Hirota *et al*, 1998; Heinrich *et al*, 2003a, 2003b; Corless *et al*, 2005). These activating mutations affect primarily the exons 9 and 11 of *KIT*, but may also be found on exons 8, 13 and 17 of *KIT* and exons 12, 14 and 18 of *PDGFRA* (Heinrich *et al*, 2003a, 2008; Debiec-Rychter *et al*, 2006). The relative frequency of the different *KIT* and *PDGFRA* mutations in patients with advanced GIST has been previously reported (Heinrich *et al*, 2003a, 2008; Debiec-Rychter *et al*, 2006). The frequency of *KIT* and *PDGFRA* mutations in localised GIST has been reported in a single-institution study from Italy. Other preliminary reports indicate that PDGFRA mutations may be higher in localised than metastatic GIST, which may reflect their more favourable prognosis. Two recent autopsy series have shown that the incidence of GIST may be as high as 50% in stomach specimens (Kawanowa *et al*, 2006; Agaimy *et al*, 2007); in one of these series, canonical *KIT* or *PDGFRA* mutations were found in 50% of assessable tumours (Agaimy *et al*, 2007). We sought to assess the precise incidence of GIST and other sarcomas in the Rhone-Alpes region in France. This report focuses exclusively on GIST.

## Patients and methods

A prospective and exhaustive study was conducted in the Rhone-Alpes region in France to assess the precise incidence of primary sarcomas and to assess conformity of management with published guidelines. Rhone-Alpes represent 10% of the French population (5 958 320 of 60 825 000 inhabitants) and 10% of the French territory. All cases for which a diagnosis of sarcoma was raised were systematically and centrally reviewed by experts (DR and AVD) to confirm the diagnosis.

### Collection of data

All pathologists of the Rhone-Alpes region (*n*=42) agreed to participate in the study after a first meeting to inform them. They had to prospectively notify their cases, and to address a paraffin-embedded tumour sample for expert review. A financial support was allocated for each notified case and each tumour bloc. Duplications were checked in the database by a comparison of the patient's initials and the date of birth. If one patient was noted to have several diagnostic materials (e.g., biopsy, surgery and metastasis), or if tumour locations were multiple, the patient could be included only once in the study.

All diagnosed patients with a first diagnosis of primary sarcoma, between 1 March 2005 and 28 February 2006, and living in the Rhône-Alpes region (as testified by the ZIP code of patients’ address), were included in the study. All subtypes of sarcomas were included: soft tissue, bone and visceral (GISTs and gynaecological sarcomas) sarcomas. Exclusion criteria concerned relapsed tumour, date of diagnosis not matched with the inclusion period, patient living outside of the Rhone-Alpes region and no definitive histology in favour of sarcoma after the review. A validation series of all new cases diagnosed between 1 March 2006 and 28 February 2007 was also collected and analysed using the same methodology.

### Pathological review

All cases of suspicious sarcomas were reviewed by regional sarcoma expert pathologists (JYS, DRV and AVD) and national experts (JM Coindre and a panel of pathologists from French Sarcoma group). New tissue sections were prepared from the paraffin-embedded samples provided by the primary pathologist, and immunohistochemical study was systematically performed again by the expert reviewer (Lurkin *et al*, 2010). Diagnoses were performed in accordance with the 2002 WHO classification.

### Mutation analysis

After manual microdissection of tumour or normal tissue, DNA was extracted from paraffin-embedded material, using the MasterPure kit (Epicentre Biotechnologies, Le Parray en Yvelines, France). Exons 9, 11, 13 and 17 of *KIT* and exons 12, 14 and 18 of *PDGFRA* were amplified using the primers detailed in Table 1.

Except for exon 9 of *KIT*, mutation screening was performed by dHPLC analysis with a WAVE system (Transgenomic, Montluçon, France). Mutations were confirmed by sequencing with the same primer as for PCR; PCR products were purified using Qiagen MiniElute PCR (Qiagen, Courtaboeuf, France) purification columns and then sequenced on both strands with the DYEnamic ET Dye terminator kit (Amersham Bioscience, Orsay, France) and analysed on a MegaBACE 1000 automatic sequencer (Amersham Bioscience).

For *KIT* exon 9, the six-nucleotide duplication was assessed using high-resolution agarose gel electrophoresis (Resophor; Laboratoire Eurobio, Les Ulis, France) of the 47 bp (or 53 bp) PCR product.

### Statistical analysis

Data were described using percentages for qualitative variables and median and range for numerical variables. All statistical analyses were performed using the SPSS 12.0 package (SPSS Inc., Chicago, IL, USA).

## Results

### Epidemiology of GIST

A total of 703 patients for whom a diagnosis of sarcoma was raised were screened; 42 patients (6.1%) were diagnosed outside the study period; 128 patients (18.5%) were found to have relapse (and not primary sarcoma); 44 patients (6.4%) managed in the Rhone-Alpes were found to live outside the predefined region; 33 patients (4.8%) lived and were managed outside the Rhone-Alpes; and 68 patients (9.8%) did not have sarcoma after expert pathologic review. In all, 376 patients (54.4%) conformed to the inclusion criteria: confirmed diagnosis of sarcoma after expert review, initial diagnosis between 1 March 2005 and 28 February 2006 (date of biopsy if there was one/of surgery if no biopsy) and resident in Rhone-Alpes region; 67 patients (17.8%) were diagnosed with GIST. For the second year, the diagnosis of sarcoma was raised in 581 patients, of whom 369 patients had a confirmed diagnosis of incident sarcoma, with 64 of them (16.7% of all sarcomas) identified as incident cases with GIST. Overall, 745 of 1284 patients had a confirmed diagnosis of sarcoma, of which 131 (18%) were GISTs. The crude incidence of GIST was therefore 11.2 per million inhabitant per year.

### Patients’ characteristics

The main characteristics of the 131 patients with GIST are described in Table 2. In brief, a majority of patients were female (*n*=75, 57%), median age was 66 (range 34–91) years and most (61%) tumours originated from the stomach. In all, 117 patients (89%) were diagnosed as having localised GIST, whereas 14 patients (11%) had metastatic disease at initial diagnosis, confined to the abdominal cavity in all cases (liver metastases *n*=9; mesentery or peritoneum *n*=8; and 2 patients had lymph node metastases). Four tumours were diagnosed incidentally on surgical specimens obtained for other reasons: two tumours were found in oesophagectomy specimens (one performed after sulphuric acid ingestion and one performed for squamous cell carcinoma), one tumour was found on a gastrectomy specimen performed for gastric adenocarcinoma and one patient had an incidental diagnosis of GIST during cholecystectomy (the surgeon resected a small gastric lesion). Risk stratification was performed according to both the National Institute of Health (NIH) (Fletcher *et al*, 2002) and the Armed Forces Institute of Pathology (AFIP) (Miettinen and Lasota, 2006) criteria (Table 2).

### Molecular data

Mutation analysis could be performed in 106 of 131 patients (81%), 55 of 67 patients (82%) in the first cohort and 51 of 64 patients (80%) in the second cohort (Table 2). In 16 cases, mutational analysis could not be performed because of the fixative used, and in the 9 remaining cases the sample size was not sufficient to retrieve enough DNA for sequencing (microbiopsies or microGIST).

The majority of samples harboured *KIT* mutations (*n*=71 of 106, 67%), and no *KIT* or *PDGFRA* mutations was found in 18 patients (17%). Seventeen tumours (16%) harboured *PDGFRA* mutations, 15 tumours originated from the stomach (14 with *PDGFRA* exon 18 and one *PDGFRA* exon 12), one from the peritoneum (omentum, *PDGFRA* exon 18 mutation) and one from the small bowel (PDGFRA exon 12 mutation). In addition, 10 patients (9%) had tumours with exon 9 mutations, 8 of which originated from the small bowel (including the duodenum), one from the rectum and one from the pelvis. The relative frequencies of *KIT* exon 11, KIT exon 9 and *PDGFRA* mutations are described in Table 3. When considering only patients with localised disease for whom molecular data were available (*n*=94), *KIT* exon 9, 11, 13 and 17 mutations were found in 9 (10%), 49 (52%), 3 (3%) and 1 (1%) patient, respectively, whereas *PDGFRA* mutations were found in 14 (15%) patients (exon 18, *n*=12 and exon 12, *n*=2), and 18 (19%) patients had *KIT* and *PDGFRA* wild-type tumours. Of the 14 PDGFRA exon 18 mutations, 13 (93%) involved codon 842, and 10 were D842V substitutions (11%). Molecular analysis could be performed for only one of the four incidentally diagnosed GISTs and showed a KIT exon 11 mutation. For the three other cases, the amount of DNA was not sufficient to perform molecular analysis.

### Correlation of mutation and risk stratification

The aim of this correlative analysis was to determine which patient would be eligible for adjuvant imatinib, and it was therefore conducted only for patients with localised disease. Furthermore, both the NIH and the AFIP risk stratification were simplified into two categories grouping very low and low-risk group on one side and intermediate- and high-risk groups (i.e., those eligible for adjuvant imatinib *per* EMEA approval) on the other. Results of this correlative analysis are depicted in Table 4. As previously described (Blackstein *et al*, 2010), approximately 15–20% of patients from the high (intermediate and high) risk group in the NIH classification were reallocated to the low (very low and low) risk group of the AFIP risk stratification. This was true for all mutations types except for PDGFRA mutants in which 50% of the patients were reclassified as low risk, leaving only 7% of patients in the high-risk group of the AFIP classification (*vs* 57% when using the NIH classification; Table 4). The distribution of PDGFRA mutant tumours between the low- and high-risk categories was significantly different from that of KIT mutant or wild-type tumours when using the AFIP classification (*P*=0.005, Kruskal–Wallis test), but not when using the NIH classification (*P*=0.452, Kruskal–Wallis test). This difference of distributions is likely the consequence of the association of PDGFRA mutations with tumours of gastric origin (Lasota *et al*, 2004).

## Discussion

This study is the first to date to report incidence with molecular analysis of GIST based on a prospectively collected exhaustive population sample (5 958 320 inhabitants in the Rhône-Alpes region). Overall, our data are in agreement with previously reported series in terms of primary tumour location, size and gender (Braconi *et al*, 2008). One of the key findings is the differences in the frequency of *PDGFRA* mutations that seem almost twice as high as that reported in patients with advanced disease (Debiec-Rychter *et al*, 2006; Braconi *et al*, 2008; Braggio *et al*, 2008; Du *et al*, 2008; Heinrich *et al*, 2008) (Table 5), including in patients with localised disease. These differences may be because of the more indolent behaviour of tumours bearing mutant *PDGFRA* (Lasota *et al*, 2004; Dematteo *et al*, 2008), whereas some KIT exon 11 and KIT exon 9 mutations have a higher risk of relapse after surgical excision. Other recently reported series have found somewhat lower frequencies of *PDGFRA* mutations: Braggio *et al* (2008) reported frequencies of 7.3% in patients with completely resected GIST in a specific population from Brazil. In both their series and ours, the small numbers preclude any definitive conclusion and these observations must be confirmed by larger studies. Emile *et al* (2009) recently reported a large series of GIST with molecular epidemiology and found that *PDGFRA* mutation were twice as high in localised GIST than that previously reported for patients with advanced disease. This series, which used different inclusion criteria (expert pathology referral), confirms what was found in our series. However, unlike Emile *et al* (2009), we could not find any significant difference in age between patients with *PDGFRA-*mutated tumours and those with other mutation type (Kruskal–Wallis nonparametric test *P*=0.295 for *PDGFRA* mutants *vs* other mutation types). Furthermore, in contrast with what Emile *et al* (2009) found, the frequency of KIT exon 9 mutations in patients with localised disease in our series was comparable to that previously reported in patients with advanced disease (approximately 10% Heinrich *et al*, 2003a; Debiec-Rychter *et al*, 2006)

Although the predictive role of *KIT* mutations have been studied and reported in patients with advanced disease (Heinrich *et al*, 2003a; Debiec-Rychter *et al*, 2006; Van Glabbeke *et al*, 2007), the precise role of mutations in the biology of localised GISTs and their correlation with prognosis remains debated, although *PDGFRA* mutations seem associated with a rather indolent course (Lasota *et al*, 2004), whereas deletions of *KIT* exon 11 encompassing codons 557 and 558 were found to have poor prognosis (Martin *et al*, 2005). More data regarding the prognostic significance of different kinase mutations will likely be generated from the control arms of the adjuvant trials that have now finished accrual (Dematteo *et al*, 2009; Gronchi *et al*, 2009).

These observations are of importance considering the recent introduction of adjuvant imatinib treatment in high-risk GIST. *PDGFRA* exon 18 mutations (D842V), which affect the activation loop of the PDGFRα kinase, are biochemically less sensitive to imatinib than the more common KIT exon 11 mutations. As they may also have a better prognosis, the molecular characterisation may prove to be useful in selecting those patients in whom adjuvant imatinib is truly required. It is noteworthy that although 57% of the tumours with *PDGFRA* mutations in our series were classified as intermediate or high risk according to the NIH consensus (Fletcher *et al*, 2002), most of these tumours were classified as low or very low risk according to the AFIP risk classification (Miettinen and Lasota, 2006). The main reason for this shift is likely the gastric origin of most of the tumours harbouring *PDGFRA* mutations (15 of 17, 88%). Similarly, the identification of exon 9 may be needed to propose the adequate dose of adjuvant imatinib (Van Glabbeke *et al*, 2007).

Finally, the absolute incidence of GIST may be largely underestimated as suggested by the reports of Agaimy *et al* (2007) and Kawanowa *et al* (2006), which reported the so-called ‘microGIST’ in up to 50% of specimens of gastrectomies performed for other causes. Conversely, most of these tumours never become ‘clinically significant’, as the rate of clinically detected GIST is 12–15 per million inhabitants in Western countries as shown by our data and others (Nilsson *et al*, 2005; Monges *et al*, 2007). It is noteworthy that three patients in the 2005 cohort and one in the 2006 cohort had a diagnosis of GIST after surgery for another cause. Furthermore, new diagnostic or imaging procedures, such as endoscopic ultrasound, may lead to an increase in the diagnosis of small GIST. The ‘true incidence’ of GIST may therefore be a moving concept in the future.

## Figures and Tables

**Table 1:** Primers used for PCR

**Exon**	**Primer**
KIT exon 9 forward	5′-GATGTGGGCAAGACTTCTG-3′
KIT exon 9 reverse	5′-TTACCTTTAAATGCAAAGTTAA-3′
	
KIT exon 11 forward	5′-CCAGAGTGCTCTAATGACTG-3′
KIT exon 11 reverse	5′-ACTGTTATGTGTACCCAAAAAGG-3′
	
KIT exon 13 forward	5′-GCTTGACATCAGTTTGCCAGT-3′
KIT exon 13 reverse	5′-GGCAGCTTGGACACGGCTTTA-3′
	
KIT exon 17 forward	5′-TGAACATCATTCAAGGCGTATTGCTT-3′
KIT exon 17 reverse	5′-TTGAAACTAAAAATCCTTTGCAGGAC-3′
	
PDGFRA exon 12 forward	5′-TCCAGTCACTGTGCTGCTTC-3′
PDGFRA exon 12 reverse	5′-GCAAGGGAAAAGGGAGTCTT-3′
	
PDGFRA exon 14 forward	5′-TGAGAACAGGAAGTTGGTAGCTCA-3′
PDGFRA exon 14 reverse	5′-GATGGAGAGTGGAGGATTTAAGCC-3′
	
PDGFRA exon 18 forward	5′-ACCATGGATCAGCCAGTCTT-3′
PDGFRA exon 18 reverse	5′-TGAAGGAGGATGAGCCTGACC-3′

**Table 2:** Patients’ characteristics

	**Year 2005**				**Year 2006**				**Overall**			
**Characteristics**	***N*=67**	**%**	**Median**	**Range**	***N*=64**	**%**	**Median**	**Range**	***N*=131**	**%**	**Median**	**Range**
Age (years)			66	34–91			64	38–88			66	34–91
												
*Gender*												
Male	25	37			31	48			56	43		
Female	42	63			33	52			75	57		
												
*Tumor location*												
Stomach	40	60			40	63			80	61		
Small bowel	15	22			21	33			36	27		
Rectum/pelvis	5	7			2	3			7	5		
Peritoneum	6	9			1	2			7	5		
Oesophagus	1	1			0	0			1	1		
												
Tumor size (mm)^a^			50	3–290			55	4–450			55	3–450
												
*Mitose count/50 HPF* a												
⩽5	42	67			36	67			78	67		
>5 and ⩽10	11	17			12	22			23	20		
>10	10	16			6	11			16	14		
												
*Disease status*												
Localised	59	88			58	91			117	89		
*NIH risk group*												
Very low	7	12			4	7			11	9		
Low	14	24			17	29			31	26		
Intermediate	22	37			19	33			41	35		
High	14	24			14	24			28	24		
NA	2	3			4	7			6	5		
												
*AFIP risk group*												
Very low	7	12			4	7			11	9		
Low	23	39			23	40			46	39		
Intermediate	11	19			8	14			19	16		
High	12	20			13	22			25	21		
NA	6	10			10	17			16	14		
												
Metastatic	8	12			6	9			14	11		
												
*Immunohistochemistry* b												
CD117 (KIT) positive	63	97			62	98			125	95		
CD34 positive	58	88			51	81			109	83		

Abbreviations: NA=not available; HPF=high power field; NIH=National Institute of Health; AFIP=Armed Forces Institute of Pathology.

a Tumour size and mitose count were available for 63 patients in the 2005 cohort, whereas size was available for 61 and mitose count for 54 patients in the 2006 cohort.

b Immunohistochemistry data were available for CD117 in 65 patients, and for CD34 in 66 patients in the 2005 cohort, and they were available for both CD117 and CD34 in 63 patients in the 2006 cohort.

**Table 3:** Results of mutation analysis

	**Year of sample collection**					
	**2005**		**2006**		**Overall**	
**Type of mutation**	***N***	**%^a^**	***N***	**%^a^**	***N***	**%**
NA	12		13		25	
Wild type	11	20	7	14	18	17
c-KIT	35	64	36	71	71	67
*Exon 9*						
Duplications	4	7	6	12	10	9
*Exon 11*						
Deletions	15	27	10	20	25	24
Missense	9	16	6	12	15	14
Insertion	2	4	3	6	5	5
Deletion and missense	2	4	5	10	7	7
Deletion and insertion	1	2	0	0	1	1
Duplications	1	2	2	4	3	3
*Exon 13*						
Missense	1	2	3	6	4	4
Exon 17	0	0	1	2	1	1
						
PDGFRA	9	15	8	16	17	16
*Exon 12*						
Deletions	2	4	0	0	2	2
*Exon 18*						
Missense	5	9	5	10	10	9
Insertion	0	0	1	2	1	1
Deletions	2	4	2	4	4	4

Abbreviation: NA=not available.

a Percentages are calculated on the number of patients for whom mutation status was available (*n*=55 for 2005, 51 for 2006 and 106 overall).

**Table 4:** Correlation of mutation type and risk using the NIH and the AFIP risk classifications for the 94 patients with both localised disease and molecular data

		**NIH**						**AFIP**					
		**Low**		**High**		**NA**		**Low**		**High**		**NA**	
	**Total** ***N***	***N***	**%**	***N***	**%**	***N***	**%**	***N***	**%**	***N***	**%**	***N***	**%**
Overall	94	30	32	60	64	4	4	45	48	41	44	8	9
													
*Kit*	62	20	32	38	61	4	6	27	44	29	47	6	10
Exon 11	49	16	33	31	63	2	4	23	47	22	45	4	8
Exon 9	9	2	22	6	67	1	11	2	22	6	67	1	11
Exon 13	3	2	67	0	0	1	33	2	67	0	0	1	33
Exon17	1	0	0	1	100	0	0	0	0	1	100	0	0
													
*PDGFRA*	14	6	43	8	57	0	0	12	86	1	7	1	7
Exon 12	2	1	50	1	50	0	0	2	100	0	0	0	
Exon 18	12	5	42	7	58	0	0	10	83	1	8	1	8
													
Wild type	18	4	22	14	78	0	0	6	33	11	61	1	6

Abbreviations: NA=not available; NIH=National Institute of Health; AFIP=Armed Forces Institute of Pathology.

**Table 5:** Comparison of the frequencies of PDGFRA mutations among series of patients depending on the setting: advanced disease *vs* population based (advanced+localised)

**Setting**	**Advanced disease—clinical trial**				**Population based**						**Population based, exhaustive**	
**Author**	**Debiec-Rychter *et al* (2006)**		**Heinrich *et al* (2008)**		**Braconi *et al* (2008)**		**Braggio *et al* (2008)**		**Du *et al* (2008)**		**Current series**	
Number of patients included	946		746		104		81		141		131	
Number analysed for mutations	377		378		94		55		141		106	
												
**Mutations**	***N***	**%**	***N***	**%**	***N***	**%**	***N***	**%**	***N***	**%**	***N***	**%**
*Kit*	315	84	314	83	81	86	40	73	108	77	71	67
Exon 9	58	15	31	8	11	12	2	4	8	6	10	9
Exon 11	248	66	275	73	69	73	38	69	99	70	56	53
Exon 13	6	2	3	1	0	0	0	0	1	1	4	4
Exon17	3	1	4	1	1	1	0	0	0	0	1	1
												
*PDGFRA*	10	3	6	2	13	14	4	7	8	6	17	16
Exon 12	1	0	0	0	3	3	3	5	0	0	2	2
Exon 18	9	2	6	2	10	11	1	2	8	6	15	15
												
Wild type	52	14	58	15	10	11	11	20	25	18	18	17
